# Watch Rochester—Give the Children a Chance

**Published:** 1909-04-15

**Authors:** Horace Fletcher


### "WATCH ROCHESTER—GIVE THE CHILDREN A CHANCE."

BY MR. HORACE FLETCHER.

An Address at West High School, Rochester, N. Y., under the auspices of the Seventh District Dental Society and the West High Social Center.

If any of those present know me at all very likely they think of me as the arch advocator of much mastication.

Some of you may associate my name with a movement called "Fletcherism", but I have come before you on this beautiful Sabbath Day in the beautiful and progressive city of Rochester, to confess another identity, and one that is the inspiring motive in all the energy I have put out in the last ten years in Europe and in this country. I must also make the confession that I am here either by accident, or by direction of the Special Providence which has been guide ing me for the past ten years, in a quest, in which, I am sure, the citizens of Rochester will join with all the force they possess when they learn what the quest is and what the nature of the emergency which demands it. I have not come to America this time by accident. I have come with a purpose so fixed that I propose to carry it with me to the grave and perhaps beyond.

I came to America this time with the intention of starting a campaign for a purpose; for a holy purpose, for a political purpose, for a purpose of decency and justice; in short, for the purpose of *giving neglected children a chance.* You may ask the question "What Children?" and I will answer, "ALL CHILDREN." Again you may ask, "Do not all children have a chance?" My answer to this question will be "*No, all children do not have a chance whatever to choose between the good and the bad.*" But I will explain myself to you in this essential regard later on. I am telling you why I came to America this time from my home in Venice, and something of the campaign I have been planning and preparing for the past ten years. It was my purpose to start the campaign in New York in the midst of what is perhaps the greatest field of need. It was my idea to give the children in greatest need the first chance. But some force has directed my steps to Rochester, and has urged me to reveal my plans, and this is my purpose in addressing you here to-day in to me, a most unexpected place, and with an inspiration that I feel to be sympathized with here especially because of the cause of its promulgator.

First of all let me say that I find here in Rochester an organization, ready made, which is adapted more perfectly than any I had planneed, to carry on the campaign that I have in mind. The idea of a communal social company to search the highways and byways, to give the last of neglected children a chance to drink of the water of life, is entirely new to me, and comes to me as a revelation illumined by the light of greatest possibilities. Mr. Edward J. Ward and Professor Geo. M. Forbes did not have to go far in their explanation of your noble organization, to let me see that a whole team of Special Providence is at work aiming to *give all the children a chance*, and that the headquarters of the team has been for the moment, pitched in your city.

Therefore, I have selected for a title to my address this afternoon the invitation—WATCH ROCHESTER—GIVE ALL THE CHILDREN A CHANCE. —

In the year 1898 I was spending some time with my family in England and had made most attractive plans for pursuing some art studies in that land of richest art and architecture. As a student of Sociology, also, I had given much attention to the subject of child-saving as the most profitable procedure in reforming social conditions on the most practical lines. A title for a book had been running in my mind for a long time and that title was, "SOCIAL QUARANTINE." The idea was a very vague one to be sure but I had been living for some years in Louisiana, where yellow fever sometimes came and ravished the country and where the necessity of strictest quarantine restrictions had been impressed on my mind. I felt that quarantine against any of the germs of human disability was the only method of prevention worth considering, and I was trying to adapt it to my chosen subject of Sociology.

One day I was confronted by a business complication in America and it was such an important matter that, on a few hours notice, I jumped on one of the fastest of trans-Atlantic Liners and hurried immediately to Chicago, passing through Hoboken only, and not crossing over to New York. At that time it was a record trip, I believe. I transacted business in London on one Wednesday and the next Wednesday I transacted business in Chicago. It was in the midst of the Spanish-American war. I had not seen the flush of American patriotism that had followed in the wake of the destruction of the "Main". The amount of patriotic display associated with business enterprise which I saw in Chicago half dazed me, coming as I had from quiet London. In walking about on the streets with a friend from New Orleans, whom I had met on arrival, I came to the portal of the great Pullman building. There I was attracted by a six-foot policeman who was shaking and cursing a mite of a boy who could not have been more than four or five years of age. The parting shot of the policeman, in pushing the infant out into the street, excited not only my interest but my pity, and I enquired of him the cause of his seeming brutality. I saw at once, however, by the tone of the voice of the big policeman, that he was no brute at heart, and that he had assumed his sternness for a purpose. The curse I had heard was, "Now git you little bastard, and to hell wid you." The policeman's explanation was something like this. I recorded from a fresh memory at the time, but I will give it more briefly here, for I have some comment to make on it besides. It seems that at that exciting time of war the receipt of news and the extravagance of street illumination in Chicago attracted immense crowds of visitors from without the city, as if at a World's fair, and they crowded the streets at night. Taking advantage of this crowding of strangers a number of gangs of slum children mixed with the country crowds and exploited the confusion of it for all it was worth. A favorite scheme of these apprentice criminals was to mix with a crowd in a candy shop; and, at a given signal, all of them "swipe" whatever of the candies they could lay their hands on, and then all rush out into the street, leaving behind them a tiny kid or two to serve as decoys for the shop people who might be intent on catching the youngsters. These young criminals well knew that the shop folk would be satisfied if they caught one of their number and would not make hopeless search in the crowd for the rest. They also knew that when the infants of their gang had been caught and turned over to the police nothing more serious than a kick and a curse would follow, for the reason that the city of Chicago had no accommodations for young offenders, either corrective or reformatory, and would have to let them go after some mild disciplining on the spot. And so it happened, and I was a witness of the finale of this criminal gang. I had also seen the face of the tiny child as it lifted in appeal to the huge policeman. It was a beautiful face and has haunted me ever since. I can scarce ever think of that face, and think of it in the light of my later enquiries into the conditions that must have surrounded that child, without the tears coming to my eyes, and I assure you I am not ashamed of emotion having such a pitiable cause. My first thought in connection with the incident was to inquire about it of the policeman. I found him as humanely feeling as I was myself, and he told me the story almost with tears in his eyes, for he knew the pity of it all and appreciated it better than I ever could do. When I had heard the story and had sympathized with the policeman on account of his inability to do any better than he had done, it all at once came upon me, like a raging desire, an impulse to find that child and give him a chance to know something of good instead of allowing him to grow up to a life of crime, forever tabooed by respectable society, without first having a chance to choose the good rather than the bad. As I walked along the Lake Front for a long time filled with the thought of my opportunity and how I had stupidly let it pass, the face of the child haunted me all the time as it is doing now. I would have given all I had in the world to have turned time backwards in its flight long enough to get hold of the little victim of our communal neglect and give him at least a fighting chance for his respectabitity. But it was of no use. The little fellow had gone into the crowd of a huge city. He might never again appear on the scene where he had been caught, but with criminal discrimination he would be used again and again in the same way as I had seen him, for foiling the arrest of the real criminals in some other section of the city. However, hopeless as it seemed, I was determmined to make the attempt to find the child. On going to bed in the Auditorium Hotel I thought long of the incident. It crowded out of my mind the business which had so urgently brought me 4,000 miles; and the excitement of war, as well, was driven entirely from my mind, and I could only think of my lost waif. But upon waking the next morning, and on listening to my waking thoughts, as is my wont, the problem of the saving of the child, and of giving him a chance to choose between the good and the bad was much cleared up; but, at the same time, it assumed such proportions that it was almost overwhelming. The vision that came first to my mind in the revelations of the morning was a bag of shot. Just a small bag of shot used in hunting for birds and small game. One of these shot had been taken from the bag and, for some reason, there was a strong desire to keep it separate from the rest of the shot in the bag. But by a slip the prized pellet dropped back into the bag and was hopelessly lost for identification. The thought that came to me at that moment was that the shot was not lost while I retained the whole bag of which it was a part, and with this thought came another—My waif was not lost. I could save him yet, but to do so I must save *all the waifs in the world,* my waif among the rest. At the same time if I had stopped to reason, the idea of saving all the waifs in the world and giving them a chance to choose between the good and the bad was preposterous. But I did not stop to reason. The only way to save the whole was to save as many as possible, and as fast as possible, without wasting time by considering the immensity of the whole aim and always with the whole and complete aim of quarantine thoroughness in view. Connected with these thoughts came another. The ghost of my SOCIAL QUARANTINE possession came up and joined the others. What was the connection? I stopped to link up the strange happenings, and I have been linking them up with unremitting fervour ever since, during a whole decade of precious time, never stopping a moment to be frightened or appalled by the immensity of the task mapped out. Neither have I pursued any predetermined plan of campaign. I have considered my waking, inspirational thoughts as messages from some source which I could not comprehend, and I have followed the same lead, not blindly but faithfully for these past ten years, and I am following it now in revealing things to you as I am doing. There is no superstition in this service. It is the reverse. It is complete reliance in the good intentions of Mother Nature in sending us messages "out of the blue," which, added to our accumulating experience, constitute true evolution. This faith is not inconsistent with any religious faith and the quest I am pursuing in the interest of neglected children is not that of any creed or cult but is a campaign to organize team-work for the purpose in view without questioning the selection of the team which Mother Nature may direct. Neither is the use of the term Mother Nature a substitute for the name of Deity by whatever name preferred. It is an effort to secure a quickening of public conscience so as to make it impossible that such horrors of condemnation as I have described can happen for long in this hospitable country of ours and not even for many generations throughout the world. It is but another spoke in the wheel of Conferences for the purpose of eliminating injustice from our civilization.

You will naturally ask why it is that my campaign has been so ineffective that in a full ten years, full half of a generation, more progress has not been made so that at least you might have heard of it.

Here is another strange example of the workings of the divine plan of evolution. But I can best explain by giving a parallel case of profitable preparedness which is known to all the world. After the Japanese Nation had learned that "might meant right" in the adjustment of international competition for power; after Japan had been robbed of the fruits of her victory over China in the interest of the advance of civilization, and when the European Powers had joined to deprive her of the over-lordship of Manchuria, it was clear to her astute statesmen that she must prepare herself quietly for the resistance of force which was appearing above her horizon, and whose intent was to drive the Japanese people into the sea, or, to take them in as subject-members of a mixed community and possess itself of the beautiful homeland of the Japanese. It was hopeless for so small a nation as Japan to cope, even handed, with so great a nation as Russia. Hence the Japanese devotion to the Bushido ideal set them to thinking how they could increase their efficiency so as to have some advantage, other than numbers, of men and of guns. They studied the weaknesses of their enemy and found that the greatest weakness of all was the unhygienic habits of their aggressive enemies. How could they cultivate more sanitary practices and secure an advantage in that manner? We have all heard what they did in this way and what they accomplished in the way of a great victory which has placed them among the most respected and envied among the great nations of the world. To me the Japanese model was particularly impressive as I had lived among the Japanese, the Chinese and the Russians of the far East for many years, and was in touch with the preparation that had led up to the great victory. Nearly thirty years before I had the pleasure of teaching the great Field Marshal Oyama, of the Japanese army, snap shooting, in order that he as minister of war, could test the efficiency of it. At that time I had qualified as an expert rifle-shot and had published a brochure on the subject of accuracy and quickness in shooting, which had met with the approval of the government, and hence my invitation to instruct the Japanese minister of war in the matter. I also gave a demonstration of personal adaption before one of the Imperial Princes, and some two or three hundred of the staffs of the army and navy. This may not be pertinent to this address, because we are occupied with saving children rather than killing men, but it is useful as an illustration. I had called my treatise on shooting "The A. B. C. of Snap Shooting," because it dealt with a method of learning to shoot that involved thoroughness of practice from the A. B. C. of the matter all the way to perfection at the end of the alphabet. I have pursued the same method all through my studies and writings, and it is the same method of thoroughness that inspires the idea of an attempt to create Social Quarantine.

But will you not ask: "What has all this to do with socialogical development?" You may well ask and I shall be glad to answer, for it explains why I dissappeared from the field of advocacy of Social Quarantine, and of giving all the babies a chance to choose between the good and the bad, to become active the world over as "The Chew Chew Man."

Co-incidently with some experiments that I had made for my own physical salvation, the story of which has been so often repeated within the past five years all over the world, came the revelation that the current teachings on the subject of nutrition were such as to poison all who followed them. It was also revealed to me as the result of the inquiry I at once set on foot that most of the human tendency to crime and sickness and lassitude, and "That Tired Feeling," was due to malnutrition. I had discovered for myself a solution of the nutrition question and that made it possible to teach the most ignorant of persons how to take their food in such a way as to prevent poisoning, and at the same time secure the maximum of benefits from their nourishment, without the collateral production of the poisons which are the cause of human disability. It is too long an explanation to follow the matter of this divine discovery through the length of its development, but suffice it to say that, coincidently with evolving a plan of thorough education, so that not a single child of the community escapes coming under the influence of it, came the revelation that a right nutrition was the basal necessity of such instruction and was especially necessary in the midst of the most civilized conditions of life where a plethora of food and false ideas of generous nutrition made it almost impossible to be hygienically temperate. The morning thought which I was following so faithfully came in at this juncture and directed that it was useless to extend the school facilities and train for these added facilities teachers who would teach poison in the form of false ideas of nutrition to the little ones who were being sought for reformation or formation. We must begin right. We must thresh out the confused problem of nutrition and come to an intelligent teaching agreement about it so that we might put the children to be taught on a sound physiological foundation. To this spiritual message of direction my finite judgment responded with avidity, as it had become accustomed to do. I said to myself, "This question of nutrition is confusion; I have solved it for myself in a manner that is teachable to the smallest of children, and the most ignorant of persons, for it deals alone with the natural instincts with which Mother Nature has equipped all of the animals, and which she has also given to her children of the great and dominant human family. These were the same natural protections that were effective long before Physiology became a Science, and before

Chemistry had a name. We could put even the most unfortunate children back into touch with their Mother Nature, and let them nourish themselves as they should, as Mother Nature required, and we could secure a habit of compliance with the natural requirements, by teaching how to make all food taste always like "That which Mother used to make." This knowledge has been revealed to me and I could easily convey it to the most respected authorities in the Science of Nutrition; they, in turn would endorse it. We would boil it down to the simplest teachable form, and have it ready for our training-schools for teachers, and for our infant schools in the slums, when we had secured their establishment, as classic rules.

It seemed so evident, so obvious, so clear, that I thought it would be easy. I determined I would go at it in a most thorough manner. I would publish a little book, and everybody could see. I would even devote a year to the preparation, so as to start right in the new plan of thoroughness, if it were necessary to do so. I was very brave, very confident and very determined. Well, instead of only one year of nutrition missionary work accomplishing my purpose it took ten years. It took three years before I received one note of credence from an authoritive source. It was ten years before Science gave it full recognition and proclaimed it the necessary basis for right instruction. At the end of that time, the American Association for the Advancement of Science made me a Fellow in recognition of "Distinguished Contribution to Science; "the trustees of the Nobel Fund gave Professor Pavlov of Saint Petersburg, $40,000 as a reward for his demonstration of the sanity of dietic righteousness as recommended. Think of a wave of foreign approval measured by more than 300 favorable newspaper and magazines notices, averaging more than a column each and backed by the technical scientific journals. With this approval and agreement we have furnished our preparations for battle against the most insiduous enemies of human efficiency and happiness and now we are ready to begin the crusade.

The Japanese took ten years to make preparations for their great struggle. It has taken us a life time. The Japanese based their hopes of superiority over overwhelmingly larger numbers on Sanitary superiority, and the caution proved justified although it involved much unusual expense. The Japanese went up against a great bubble of power filled with ignorance, and so are we going out to meet a similar enemy. The Japanese accomplished victory in about two years. So can we kill ignorance in the same time with the facilities for publicity which we possess. We may not conquer the world of ignorance in two years but we can cure all the ignorance within the city limits of Rochester within that time if there is sufficient concentration of the people who know.

It is not a question of great expense, as in the case of a war with guns and high explosives against strong and determined men. It is a war against bad teaching and neglect among infants, and there is opposition. It is war against continuing the teaching of poisonous suggestions and thereby allowing nature to have a chance to express herself normally. It is not so much a war of aggression as a surrender to a better power whose intentions are the best for us. It is a plan to have the expense of sustenance, secure immunity from disease, double the working energy kill an unsanitary tendency to morbid sexuality and alcoholic intemperance, and the establishment of a complete and thorough quarantine against the miserable, costly unprofitable and killing enemies of human health and happiness.

It was my intention to form an organization and start a campaign in New York, mapping off a section of the worst quarters of the city for isolation and experiment and creating thereby a model of reform possibilities on a physiological basis for the rest of the city, for the rest of the country, and for the rest of the world. But providence has made other plans for us. I have been drawn here from my home in Venice by the insistence of the most energetic dental council I have ever encountered to deliver an address on the mental and dental departments of the conservation of health and energy, and I find a ready-made organization and a ready made-enthusiasm, and a completely ready-made equipment for creating a model here in Rochester.

Rochester is as well known over the world as almost any city having a name. The diverse manufacturers of Rochester have carried the name far and wide and favorably all over the world. I have been reminded of it in India, China, Japan, Java, the Phillippines and even in such remote places as the Vale of Cashmere and in savage Borneo, but it is a culmination of the great and potent unexpected to find three great factors of assistance in creating a model of reform on a physiological basis in a most favorable field of experiment, in a progressive and responsive university, and in a Mechanics Institute conducting an immense school. That is the way of Providence when you give her a chance.

Fletcherism believes in giving Providence a chance all along the line, but first in *giving all the babies all the chance possible all the time* until they can manage Providence intelligently themselves; of domestic science in a spirit of athletics in an organized scheme of commercial get-togetherness, and in a half dozen men whom I have met who are such wheel-horses of civic altruism that anything they will to do and anything so simple as what I propose to do that they approve is the same as accomplished when they have made up their minds, to have it done. I can see as clearly as I can see light, that my Social Quarantine ideals will find accomplishments here and now before very long and that the idea will spread over the world. In the meantime, with the publicity facilities which are freely offered us in this propaganda we will hoist the invitation as expressed in my title.

I will say by way of conclusion that I am not building theories on mere hopes. The completeness of child salvage which I propose has been demonstrated possible and the value of putting infant training for good citizenship on sound physiological basis has been proven. I will not say we have exhausted experiment and acquired all the knowledge we can secure.

I have met a suggestion relative to detail of procedure here that is of epoch-making value. Mr. Ward, the enthusiastic leader in your social community idea uttered the suggestion referred to just now, last evening at the Club, and it was so brilliant as to illumine a whole field of doubt with success.

As a result of the revelation of civic altruism which have come to me here in Rochester, I feel that whatever good there may be in my simple suggestions will be eagerly taken up and applied better than I could plan myself. I claim nothing for the ideas evolved and expressed, but the happy agency of being entrusted with those by our common Mother Nature for delivery to all her children even unto my waif of the Chicago incident, and to *that last one of all the waifs*, allowing no one of them to escape.

Thoroughness spells ease. Thoroughness is the aim of Fletcherism. Thoroughness is the secret of Japanese success. Thoroughness is the kernel of Japanese Bushido. Thoroughness is easy, but lack of thoroughness is always hard. Thoroughness of voluntary digestion insures thoroughness of involuntary digestion. Thoroughness of dental care insures ease of voluntary digestion. Thoroughness of child care insures universal good citizenship. Thoroughness of child care for insignificant cost, while child neglect among the hopelessly submerged costs the country untold millions in correctional expense and lack of productive energy. Thoroughness means beginning at the beginning and working for aid. It means paying first attention to the health of the individual in the beginning of the life of the individual. Fletcherism stands for chewing and for giving all the babies a chance. This then is the beginning and the end of thoroughness.

I have revealed to you some possibilities of practical reform for which you have an especial equipment already made, and as far as I can judge, quite complete. If you see good in any of my suggestions I am at your service with all my facilities.

I may say I have been entrusted with some planning for the work of Chautauqua next season that will astonish the world. It is in the line of further demonstration of the great natural possibilities of increased health and efficiency based upon dietetic righteousness. Any effort you may make will be beautifully assisted there and among the more than six hundred Chautauquas which take cue from Chautauqua on the Lake.

Will you enthusiastic citizens of Rochester accept the call? Providence has blessed you greatly and she wants to bless you more.

